# Altered spatial summation optimizes visual function in axial myopia

**DOI:** 10.1038/s41598-020-67893-8

**Published:** 2020-07-22

**Authors:** Victoria Stapley, Roger S. Anderson, Kathryn J. Saunders, Pádraig J. Mulholland

**Affiliations:** 1 Centre for Optometry and Vision Science, Biomedical Sciences Research Institute, Ulster University, Cromore Road, Coleraine, BT52 1SA Northern Ireland UK; 2 National Institute for Health Research (NIHR) Biomedical Research Centre at Moorfields Eye Hospital NHS Foundation Trust and UCL Institute of Ophthalmology, London, UK

**Keywords:** Retina, Medical research

## Abstract

This study demonstrates significant differences between the area of complete spatial summation (Ricco’s area, RA) in eyes with and without non-pathological, axial myopia. Contrast thresholds were measured for six stimuli (0.01–2.07 deg^2^) presented at 10º eccentricity in 24 myopic subjects and 20 age-similar non-myopic controls, with RA estimated using iterative two-phase regression analysis. To explore the effects of axial length-induced variations in retinal image size (RIS) on the measurement of RA, refractive error was separately corrected with (i) trial lenses at the anterior focal point (near constant inter-participant RIS in mm), and (ii) contact lenses (RIS changed with axial length). For spectacle corrected measurements, RA was significantly larger in the myopic group, with a significant positive correlation also being observed between RA and measures of co-localised peripheral ocular length. With contact lens correction, there was no significant difference in RA between the groups and no relationship with peripheral ocular length. The results suggest RA changes with axial elongation in myopia to compensate for reduced retinal ganglion cell density. Furthermore, as these changes are only observed when axial length induced variations in RIS are accounted for, they may reflect a functional adaptation of the axially-myopic visual system to an enlarged RIS.

## Introduction

Myopia is a common refractive condition, whereby the axial length of the globe is too great for its optical power. Whilst the optical refractive error of myopia can be corrected using spectacles or contact lenses, the axial elongation of the myopic eye can markedly increase the risk of sight-threatening conditions such as retinal detachment^1^, glaucoma^2^, and myopic macular degeneration^3^. In the absence of such pathological processes it has also been demonstrated that the globe elongation that occurs in myopia can lead to secondary peripheral retinal thinning^4–6^, in addition to a reduction in the density of both photoreceptors^7–9^ and retinal ganglion cells (RGCs)^10,11^.

Deficits in visual function have also been reported in the myopic, but otherwise healthy, visual system. Numerous studies have objectively investigated retinal function in myopia through measurement of standard electroretinograms (ERG)^12,13^ pattern ERG^14^ and multifocal ERG^4,13,15^. These studies have revealed altered responses in myopes, including reductions in amplitude^12–14^ and longer implicit times^4,13,15^. Other studies have reported reductions in function when examined using clinical tests of visual acuity^16,17^, peripheral resolution acuity^4,18,19^, and contrast sensitivity^20^.

It may be hypothesized that changes in visual function observed in non-pathological myopia may be accounted for by reductions in the local density of retinal neurons (e.g., RGCs) and corresponding alterations in the basic visual process of spatial summation. This refers to the ability of visual system to integrate light energy over area and serves to maximize the detection of a signal in the presence of visual noise. Spatial summation is governed by Ricco’s law, this stating that for stimuli of sufficiently small area summation is complete, with the product of stimulus area and contrast at threshold being constant^21^. Ricco’s area (RA) is the largest area for which complete spatial summation occurs, with incomplete summation being exhibited for stimuli larger than RA. The size of RA has been shown to increase in the healthy visual system with retinal eccentricity^22–26^, and reduced background illuminance^27–29^, as well as in some forms of ocular disease such as glaucoma^30–32^. It has been hypothesized that such dynamic changes may serve as a mechanism to maintain the input of a constant number of functional RGCs to cortical receptive fields, thus ensuring a constant sensitivity in the presence of visual noise^24,31,33,34^. We hypothesise that similar changes in spatial summation are likely to occur in non-pathological myopia to compensate for reduced RGC density secondary to ocular growth and retinal stretch.

Two previous studies have investigated spatial summation in myopia. Jaworski et al.^35^ restricted their measurements to the foveal region only, comparing emmetropes to high myopes (mean refractive error −10D). The authors observed a 55% and 43% increase in the size of what was defined as the ‘critical area at maximum summation’ in myopia for S-cone and achromatic stimuli respectively. However, the increase noted for the achromatic stimulus failed to reach statistical significance, likely due to the small sample size. Spatial summation was subsequently measured by Atchison et al.^19^ for a larger cohort of myopes, with refractive errors ranging from −0.50D to −12.5D, at a range of visual field eccentricities from 0 to 30 degrees along the horizontal meridian. An increase in RA was observed at the fovea and in the temporal visual field in myopia, but no significant changes were observed nasally. Both studies however used a constrained fitting technique, whereby the slope of the first and second lines in a bi-linear summation function were fixed, assuming either complete or a fixed degree of partial or no summation, this method being known to bias estimates of RA^36^. In addition, neither study investigated the effect of prospectively controlling axial-length induced alterations in retinal image size (RIS) on measures of spatial summation despite the fact that RIS is larger in axial myopes compared to emmetropic or hyperopic observers. Indeed, it has been proposed that a ‘neural minification’, occurring secondary to an increased spacing of retinal elements and possibly reflective of altered spatial summation, likely accounts for an enlarged RIS in axial-myopia and may serve to optimize visual function in myopic observers^37,38^. Considering this, we propose that the presence of altered neural processing in the myopic visual system may only be manifest when a constant inter-observer RIS, which is independent of axial length, is employed. To-date no study has investigated this.

The purpose of this study was to determine if RA is enlarged in non-pathological axial myopia and to quantify the relative contribution of local neural elements (e.g., RGC layer thickness, RGC number) to measures of spatial summation. The effect of higher-order aberrations and axial length induced differences in RIS on spatial summation was also investigated with a view to isolating optical and neural induced changes on this neurophysiological process.

## Methods

### Participants

Twenty-four participants with axial myopia (mean 26.9, range 18–58 years) and twenty age-similar non-myopic controls (mean 26.4, range 19–53 years) were recruited for this study. All participants had a best corrected Snellen visual acuity of 20/20 (6/6) or better in both eyes, astigmatism < 1.50DC in the test eye, no visual field defect measured with the 24–2 SITA standard threshold test (Humphrey Visual Field Analyser, Carl Zeiss Meditec, Dublin, CA) and intraocular pressure ≤ 21 mmHg as measured using Goldmann applanation tonometry. Peripapillary retinal-nerve-fibre-layer (RNFL) scans also revealed RNFL thickness to be within normal limits and macular OCT scans revealed no abnormalities (Spectralis OCT, Heidelberg Engineering Gmbh., Heidelberg, Germany). A clinical examination identified no media opacities or concurrent ophthalmic disease, and participants did not have any systemic conditions or take any medications that could affect vision.

Refractive error was measured objectively in each participant using a binocular open-field autorefractor (Shin Nippon NVision-K 5001, Japan) following the instillation of Tropicamide Hydrochloride 1.0%. Participants fixated on a Maltese cross target positioned on a flat wall at a distance of six meters, with an average of three measures being taken. Myopia was defined as a spherical equivalent refractive error ≤ −0.50DS^39^. The myopic group had central refractive errors ranging from −0.50DS to −9.75DS (mean −4.14 DS), with refractive errors ranging from −0.25DS to +1.75DS (mean +0.71 DS) in the control group. Based on the World Health Organisation (2015) definitions, nine participants were defined as having high-myopia (≤ –5.00DS), with the remainder (n = 15) having myopia in the range −0.50DS to −4.75DS (low-moderate myopia). The characteristics of each group, along with biometric measurements, are displayed in Table 1.

**Table 1:** Characteristics of the myopic and control groups.

	Controls (n = 20)	Myopes (n = 24)	Low-moderate myopes (n = 15)	High myopes (n = 9)
Age (years)	22.50 (20.00 to 31.00)	23.00 (20.00 to 28.50)	22.00 (19.50 to 28.00)	23.00 (22.00 to 27.00)
Refractive error BVS (DS)	+0.50 (0.00 to +1.25)	−3.63 (−2.00 to −6.00)	−2.50 (−1.75 to −3.75)	−7.00 (−5.63 to −7.88)
Astigmatism (DC)	−0.25 (0.00 to −0.75)	−0.50 (0.00 to −1.00)	−0.50 (0.00 to −1.00)	−0.50 (0.00 to −1.00)
Axial length (mm)	23.64 (23.01 to 24.01)	25.20 (24.56 to 26.00)	24.61 (24.24 to 25.41)	26.33 (25.64 to 27.95)
Anterior chamber depth (mm)	3.60 (3.45 to 3.86)	3.73 (3.53 to 3.90)	3.72 (3.52 to 3.80)	3.93 (3.62 to 4.05)
Average corneal curvature (mm)	7.91 (7.81 to 8.07)	7.79 (7.62 to 7.91)	7.84 (7.67 to 8.00)	7.64 (7.41 to 8.14)

Summary values are presented as median (IQR).

This study received ethical approval from the Ulster University Biomedical Sciences Research Ethics Filter Committee and the research adhered to the tenets of the Declaration of Helsinki. Informed, written consent was given by all subjects prior to data collection.

### Refractive correction

For all participants, refractive correction was achieved by (i) full aperture trial lenses placed at the anterior focal point of the eye (15.2 mm) such that Knapp’s law, minimizing relative spectacle magnification, was satisfied (i.e., RIS equal to that in an emmetropic eye was maintained for all participants with varying axial ametropia) and, (ii) soft contact lenses where Knapp’s law was not satisfied (i.e., RIS was not equal with varying axial ametropia)^40^. The power of trial lens for correction was determined by non-cycloplegic objective refraction (Shin Nippon NVision-K 5001 binocular open field autorefractor, Shin-Nippon, Tokyo, Japan) and subjective refraction at a 6 m viewing distance. For all experimental tests an appropriate, subjectively refined near addition was incorporated to account for reduced accommodative facility post pupil-dilation and the monitor viewing distance. The correct back vertex adjustment was made for refractive errors ≤ −4.00DS when calculating the power of contact lens correction to use. The order in which participants undertook the spectacle corrected and contact lens corrected measurements of spatial summation was randomized to minimize any bias due to learning effects or fatigue. Refractive correction was provided to the test eye only, with the fellow eye occluded using an opaque eye-patch.

### Apparatus and stimuli

All stimuli were presented on a gamma-corrected CRT display (SONY 420GS; Sony Corp., Tokyo, Japan; pixel resolution, 1,280 × 965, refresh rate 75 Hz, viewing distance 620 mm) after a 1.5 h warm up period. The achromatic background had a mean luminance of 10 cd/m^2^ and the maximum luminance of the test stimuli was 126.6 cd/m^2^. The chromaticity co-ordinates of both the background and stimuli were x = 0.258 and y = 0.257 as measured using a colorimeter (ColorCAL-II, Cambridge Research Systems, Rochester, UK). Stimuli were generated using MATLAB (2016b, The MathWorks Inc., USA) with Psychtoolbox (v3.0) and a Bits-# (Cambridge Research Systems, Rochester, UK). Participant responses were collected using a Cedrus RB-540 response pad (Cedrus Corporation, San Pedro, CA).

Experimental measurements were either completed on the same day as the screening tests, or on a separate day depending upon individual preference. All experimental measurements were carried out on one eye only with the pupil of the test eye being dilated with Tropicamide Hydrochloride 1.0% to maintain a constant photopic inter-observer retinal illuminance. Contrast thresholds for six, achromatic, circular stimuli of area ranging from 0.01–2.07 deg^2^ and Bridgeman^41^ duration 187.8 ms (15 frames) were measured at four peripheral locations at 10º eccentricity (along 90º, 180º, 270º and 360º meridians). Participants were asked to fixate on a central cross target throughout all measurements. To account for spatial luminance inhomogeneity of the CRT display, localized contrast thresholds were determined for each test location using luminance values for the background and stimulus measured at each test location using a colorimeter (ColorCAL-II, Cambridge Research Systems, Rochester, UK).

To determine if higher order aberrations (HOAs) influence measures of spatial summation these were measured using an aberrometer (Imagine Eyes irx3 Wavefront Aberrometer, France) in the test eye, post dilation, both with and without a contact lens in situ. All measures were captured immediately post-blink such that habitual tear film and optical quality were reflected in the measures. Accurate alignment between the pupillary plane of the eye and the instrument lenslet array was obtained through the adjustment of an internal graticule over the pupil and focusing of the Purkinje images. The participant was asked to fixate on the internal target, a black 6/12 (20/40) letter ‘E’ on a white background. Three measurements were taken under each condition and an average obtained. HOAs were analysed over a 6-mm pupil using Zernike polynomials (ZPs) from third to sixth order. The root mean square (RMS) of the total HOAs (3rd–6th order ZPs) was used in further analysis.

### Psychophysical procedure

Contrast thresholds for the six achromatic stimuli were determined using a randomly interleaved 1–1 ‘YES–NO’ staircase procedure, with a 0.05 log unit (0.5 dB) step size. Each stimulus area was considered in a separate run in a randomized order, with thresholds for the four locations being measured within each stimulus run in a randomly interleaved fashion. Each staircase terminated after six reversals with the threshold being calculated as the mean of the final four reversals. False positive rate was monitored using the presentation of stimuli of 0% contrast, with tests being rejected and repeated if the false positive rate was above 20%. Following each stimulus presentation, a listening window of two seconds for the collection of participant responses was permitted. If, following the closure of this listening window, no response was collected the stimulus was assumed to be unseen.

### Structural measurements

Co-localized structural measures of peripheral ocular length and retinal-ganglion-cell-layer (RGCL) thickness were obtained following the instillation of Tropicamide Hydrochloride 1%. Peripheral ocular length measurements were captured using an IOL Master (Carl-Zeiss Meditec, USA). A custom-built four-LED ring target was affixed to the front of the instrument to allow peripheral measurements at 10° along the four primary meridians. Three measurements were taken at each position, with an average peripheral ocular length being calculated for each participant. Possible confounding effects of ocular rotation on measurements of peripheral ocular length were presumed insignificant due to the small eccentricity measured and short duration of eccentric fixation required to obtain the measurement.^42,43^ Previous work has also reported that the IOL-Master is capable of repeatable and reliable off-axis measurements up to 40° eccentricity^44^.

RGCL thickness values were obtained by taking a 24° × 24° posterior pole scan centred on the fovea with the Spectralis OCT (Heidelberg Engineering Gmbh., Heidelberg, Germany). Participant mean keratometry values were input to minimize the effects of inter-individual variations in ocular magnification on transverse measures captured^45^. An 8 × 8 grid was then centred over the fovea with any errors in the automated segmentation being manually corrected. Mean RGCL thickness across the measurement grid squares (3° × 3°) within which the corresponding locations examined in the visual field fell (after correction for retinal ganglion offset from underlying photoreceptors^46^) was used to examine the relationship between functional measures and underlying retinal structure.

The number of RGCs underlying RA in each observer was also estimated using two methods in this study. In method one histological RGC counts from an age-similar cohort^47^ were used to produce normative values of RGC/mm^2^ over the central retina (4 mm eccentricity). These values were subsequently scaled to simulate a global expansion (‘balloon’) model of myopia, whereby RGC density proportionally changed for axial length values that departed from that expected in an emmetropic eye (23.3 mm)^48^, assuming a constant number of RGCs. The number of RGCs underlying a given stimulus area was subsequently calculated as the product of the mean histologically derived RGC/mm^2^ values over the area of stimulus presentation and stimulus area in mm^2^ (histology method, RGC_Hist_). The second method utilized the technique described by Raza and Hood^49^ to infer the RGC number underlying a stimulus in a given observer from OCT data (RGC_OCT,_ Eq. 1). In short, this used OCT derived RGCL thickness (mm) in a given observer (RGCL), co-localized stimulus area (S_area,_ mm^2^), and normative RGC volumetric density (RGC/mm^3^) of RGCL tissue (GCD, calculated by dividing the mean RGC/mm^2^ across the area of the stimulus extrapolated from unscaled, age-similar histological data^47^ with co-localized OCT derived RGCL thickness [mm] values in healthy, non-myopic observers).

1 $${\mathrm{R}\mathrm{G}\mathrm{C}}_{OCT} = RGCL\cdot GCD\cdot {\mathrm{S}}_{area}$$

For all calculations, an observer specific conversion factor (q_p_) was calculated using the abbreviated axial length method^50^ to translate degrees of visual space to mm on the retina at the test eccentricity. This value was a constant when considering spectacle corrected data, and proportional to axial length with contact lens correction in this study. Further details on both models to estimate RGC number are available in the Supplementary materials.

### Statistical analysis

For each participant, an average contrast threshold for each stimulus size was calculated across the four peripheral locations, a spatial summation function then being plotted using these average values. In the case of the contrast threshold at a given location being greater than the maximum output of the display monitor used (ceiling effect) these data were excluded from analysis. Summation functions were fit using iterative two-phase regression analysis where the slope of the first line in the function was constrained to −1 (reflecting complete summation), but the slope and intercept of the second line (representing partial summation) was free to vary. The intersection of the two lines was taken as the upper limit of complete summation or RA. Data were excluded from further analysis if the bilinear model had a poor fit (R^2^ < 0.9), or if RA was smaller than the smallest stimulus used. If the estimated RA value was greater than the largest stimulus used, RA was taken to be the largest stimulus area.

To investigate the relationship between the size of RA and co-localized ocular length and RGCL thickness measures, Passing-Bablok regression (transformation method) was used. This technique was chosen as it is suitable for a non-parametric data set, permits error in both the x and y variables, is less influenced by the presence of outliers and has been demonstrated to yield more precise estimates of slope and intercept compared to ordinary least squares or Deming regression^51,52^. A central assumption of this analysis is that the relationship between the x and y variables is linear. This was tested using a cumulative sum (cusum test), with a null hypothesis that the variables are linear. The other prior assumption is that there is a significant positive correlation between the two variables, as determined by Kendall’s tau correlation^53^. If a significant, positive, linear correlation exists, then a regression line was plotted using the Passing-Bablok procedure. For all analyses, the strength of any correlation was obtained with Kendall’s tau correlation coefficient where a linear relationship between variables was demonstrated with a cusum test.

Statistical analysis was carried out using MATLAB (2019a, The MathWorks Inc., USA) and R (Version 3.6.2). For all statistical tests an alpha of 0.05 was considered statistically significant, with Holm-Bonferroni correction applied where indicated. In all cases a Shapiro–Wilk test was used to determine if data sets followed a normal distribution and the appropriate parametric or non-parametric statistical tests were applied accordingly.

## Results

### Contribution of axial elongation to refractive error

To determine if Knapp’s Law may be invoked in the study cohort, and thus ensure that only neural contributions to RA were investigated, it was necessary to demonstrate that the refractive error of participants was axial in origin. This was achieved by calculating the spherical equivalent refractive error from measures of axial length assuming the ametropia was solely axial in origin (D_P_, based upon the method of Chui et al.^18^ using the Bennett and Rabbetts three-surface schematic eye^54^, Eq. 2 where AL = axial length in mm) and comparing these estimates with ground truth values (D_Obs_, objectively measured refractive error) for the whole study cohort (i.e., myopes and non-myopes).

2 $${\text{D}}_{{\text{P}}} \, = \,{1}.{53}*\left( {{1}/\left[ {{\text{AL}}/{1}000} \right]} \right) - {63}.{8}$$

**Figure 1:**
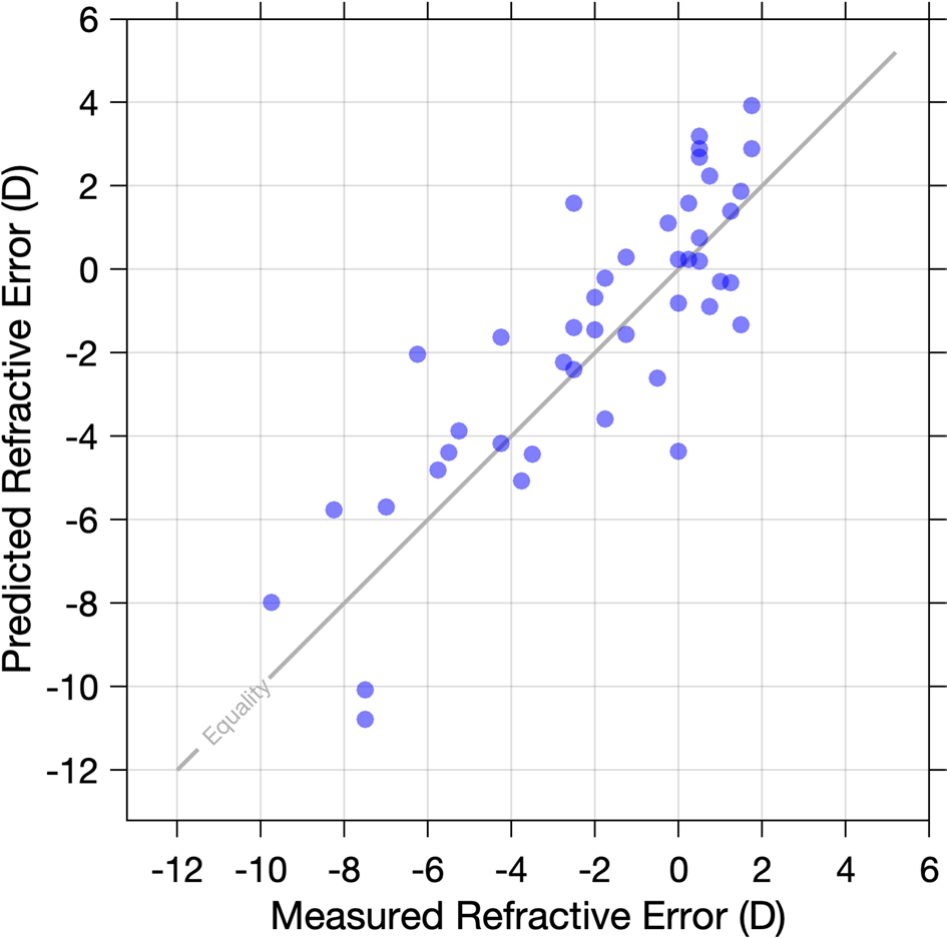
Plot of predicted refractive error (based on all refractive error being axial in origin) and objectively measured refractive error. The line of equality (yellow) is included for reference.

Spearman’s rank correlation analysis revealed there to be a strong and statistically significant relationship between the estimated and predicted refractive error values (rho = 0.81, *P* < 0.001, Fig. 1). No statistically significant difference between the measured and predicted refractive error values were also observed when examined using a Wilcoxon-Signed Rank test (*P* = 0.12).

### Higher-order aberrations

Unaided, no significant differences in the Root Mean Square (RMS) for total HOA were observed between the myopia and control groups (control: mean 0.33 µm ± 0.12; myopia: mean 0.34 µm ± 0.10, unpaired t-test *P* = 0.95). In addition, no significant relationship existed between RMS values and either refractive error (Kendall’s tau = 0.08, *P* = 0.46) or axial length (Kendall’s tau = −0.13, *P* = 0.20). For both study groups, the mean RMS for total HOA increased with the contact lens in situ (control: mean 0.36 ± 0.13; myopia: mean 0.39 ± 0.10), but this increase was only found to be statistically significant for the myopic group (myopia: *P* = 0.02; control: *P* = 0.23, paired t-test). There were no statistically significant differences in HOAs between the myopia and control groups with contact lenses in situ (*P* = 0.36, unpaired t-test), and no significant relationship between HOA with contact lenses and either axial length (Kendall’s tau = −0.04, *P* = 0.70) or refractive error (Kendall’s tau = −0.02, *P* = 0.89).

### Spatial summation in myopes vs non-myopes

For spectacle corrected measurements, an average peripheral RA value was obtained for all participants in the myopia group and 19 out of 20 participants in the control group (one control participant was excluded as RA was smaller than the smallest stimulus examined). For contact lens corrected measurements, an average peripheral RA was obtained for 23 out of the 24 participants in the myopia group (one participant excluded as R^2^ < 0.9 for fitted summation function) and all control observers.

**Figure 2:**
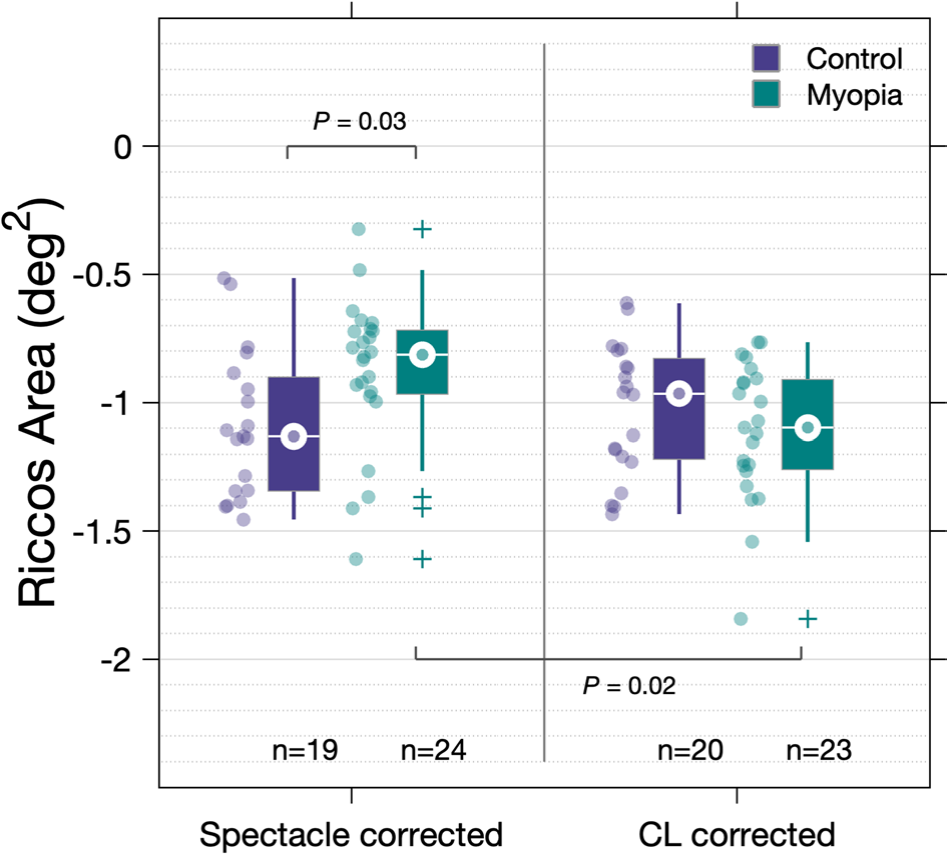
Average peripheral Ricco’s area measured for myopes and controls with spectacle and contact lens correction. Spot markers representing data from individual observers are included for reference.

**Figure 3:**
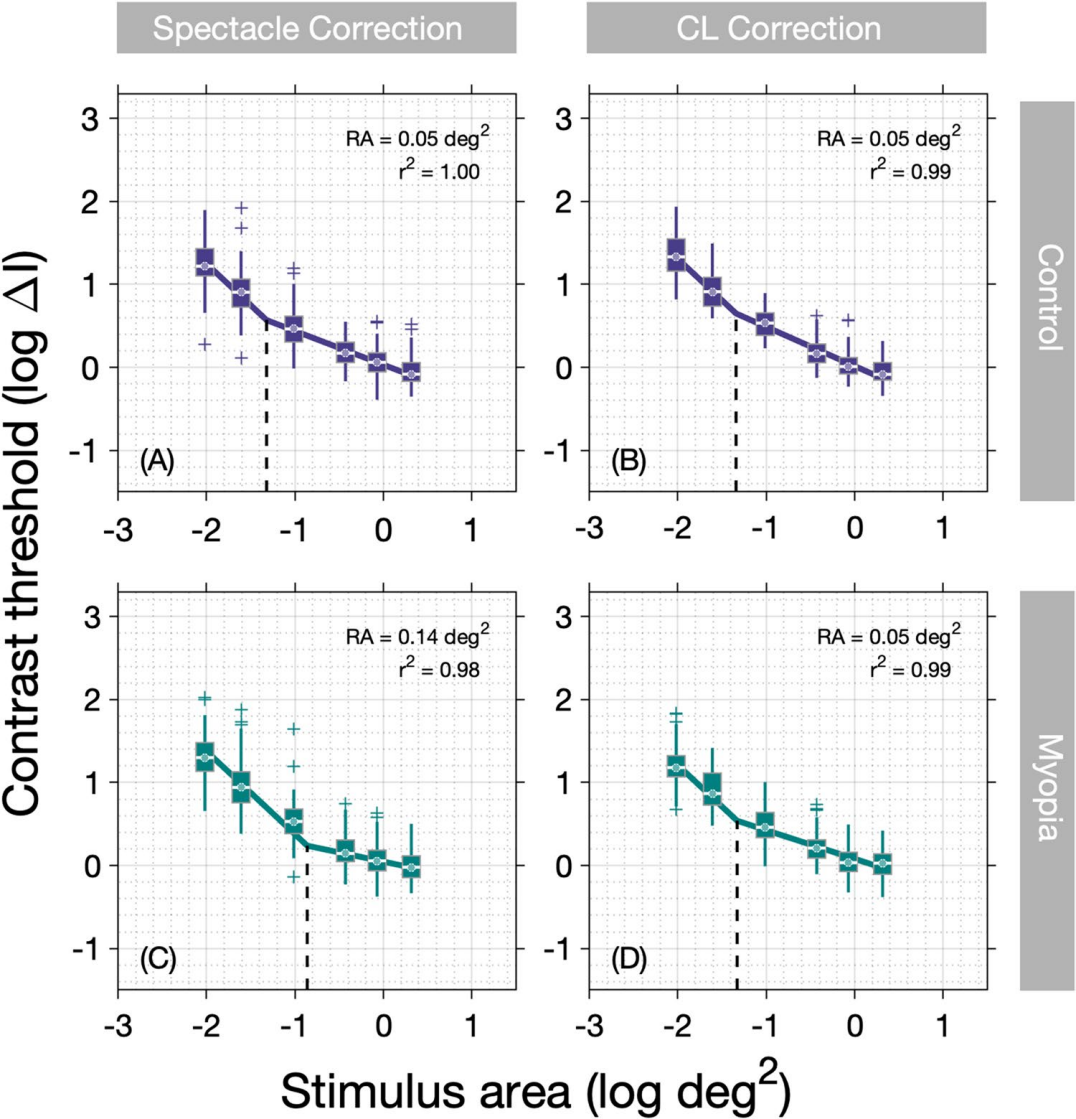
Summary spatial summation functions constructed using median thresholds for **(A)** controls—spectacle corrected, **(B)** controls—contact lens corrected, **(C)** myopes—spectacle corrected, and **(D)** myopes—contact lens corrected.

For spectacle corrected measurements, median RA was significantly larger (*P* = 0.03, Mann Whitney U-test) in the myopia group (−0.81 log deg^2^, IQR −0.97 to −0.72) compared to the control group (−1.13 log deg^2^, IQR −1.34 to −0.88). For contact lens corrected measurements, no significant difference (*P* = 0.42, Mann Whitney U-test) was observed between the myopia (−1.10 log deg^2^, IQR −1.27 to −0.91) and control groups (−0.97 log deg^2^, IQR −1.22 to −0.83). Data are displayed graphically as boxplots in Fig. 2 and summary summation functions (using median thresholds) in Fig. 3. When comparing spectacle and contact lens measures for the same individual, RA was found to be significantly smaller in the myopia group when corrected with CL compared to spectacles (*P* = 0.02, Wilcoxon signed-rank) (Fig. 2). In contrast, no significant difference in RA was observed for the controls when measured with contact lenses and when measured with spectacles (*P* = 0.62, Wilcoxon signed-rank).

**Figure 4:**
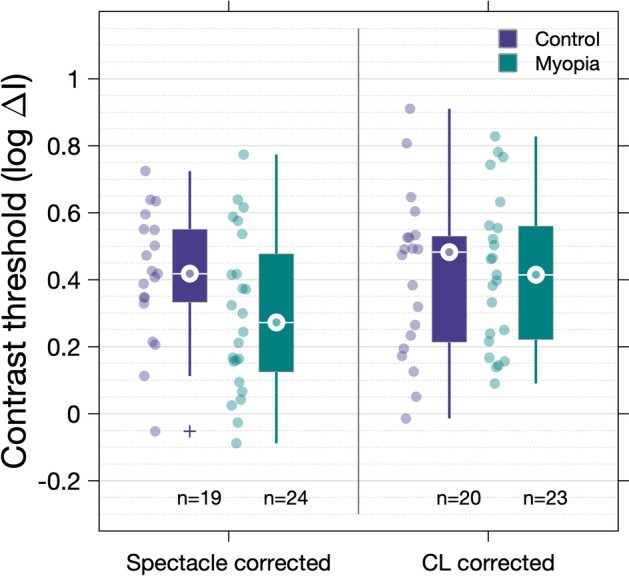
Contrast thresholds for a stimulus equal to Ricco’s area in the control and myopia groups as measured with spectacle and contact lens correction.

Interestingly, the contrast at threshold for a stimulus equal to RA was found to be lower in the myopia group (median 0.27 log ∆I, IQR 0.12–0.53) compared to controls (median 0.42 log ∆I, IQR 0.12–0.48) for the spectacle corrected data; this difference however failed to reach statistical significance (*P* = 0.15, Mann Whitney U-test, Fig. 4). No difference in the threshold at RA was observed between the groups when the contact lens corrected data were examined (Myope median 0.41 log ∆I, IQR 0.22–0.56; Control median 0.48 log ∆I, IQR 0.21–0.53; *P* = 0.99, Mann Whitney U-test, Fig. 4).

### Relationship between Ricco’s area and structural measures

**Figure 5:**
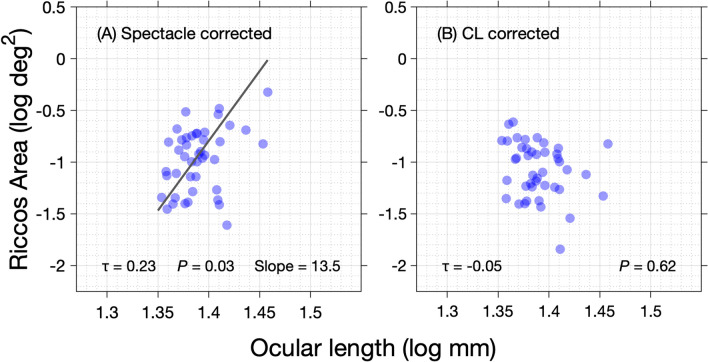
Peripheral Ricco’s area plotted as a function of peripheral ocular length for **(A)** spectacle corrected measurements and **(B)** CL corrected measurements.

For spectacle corrected measurements, a weak yet statistically significant positive linear relationship was observed between peripheral RA and corresponding peripheral ocular length values (Kendall’s tau = 0.23, *P* = 0.03, Fig. 5A). Passing-Bablok regression revealed that RA (log deg^2^) increases by a factor of 13.5 per log unit increase in co-localized peripheral ocular length. For the contact lens corrected measurements, no significant relationship between peripheral RA and co-localised peripheral ocular length was observed (Kendall’s tau = −0.05, *P* = 0.62, Fig. 5B).

**Figure 6:**
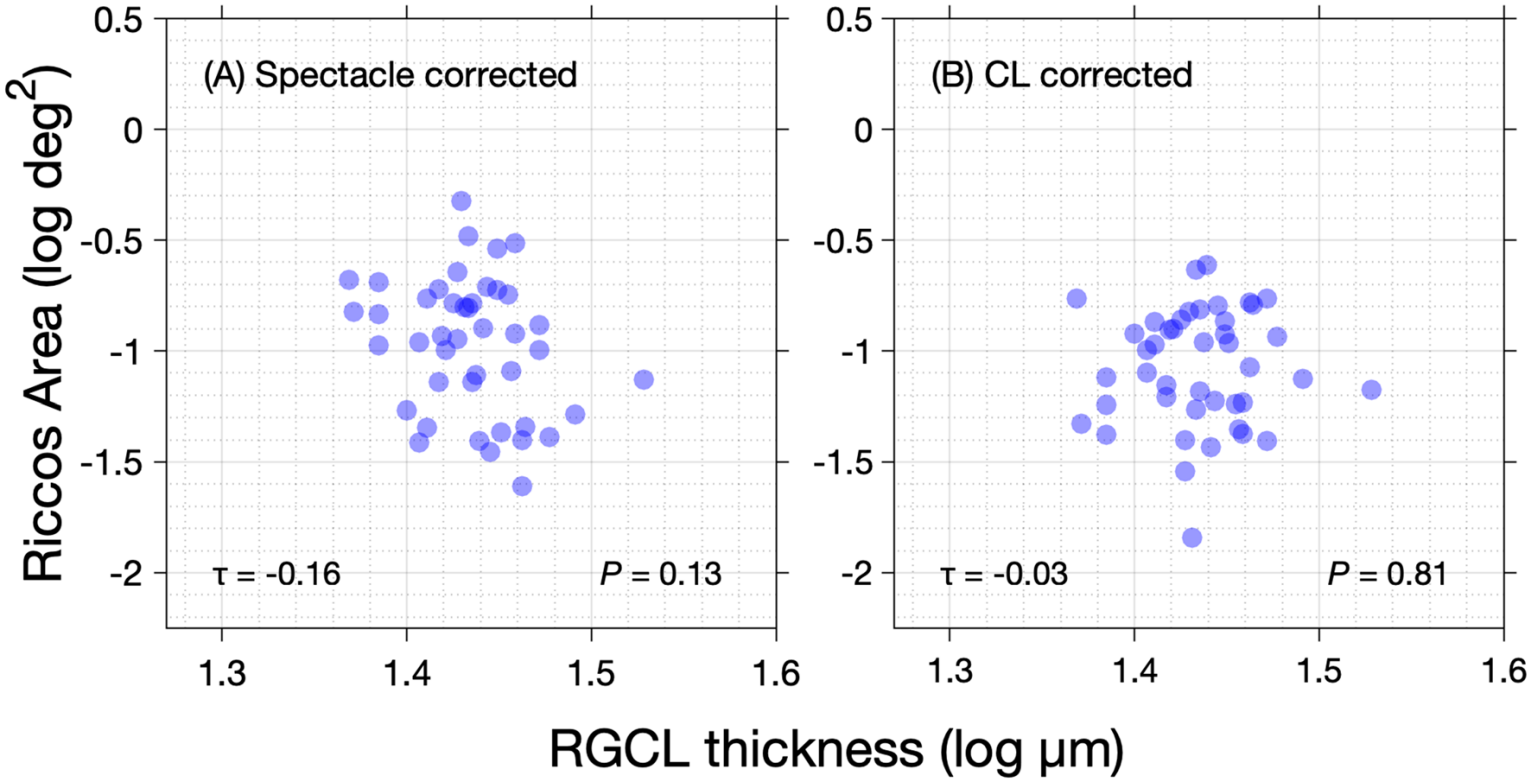
Peripheral Ricco’s area plotted against peripheral RGCL thickness for **(A)** spectacle corrected measurements and **(B)** CL corrected measurements.

Mean peripheral RGCL thickness was significantly thinner in the myopia group compared to the controls in the locations examined (*P* < 0.01, unpaired t-test). There was also a significant (p = 0.02) negative relationship (Kendall’s tau = −0.24) between mean peripheral ocular length (log mm) and mean peripheral RGCL-thickness (log µm). When considering the relationship between peripheral RGCL thickness and spectacle-corrected RA, a weak, negative correlation was observed (Kendall’s tau = −0.16). This relationship however failed to reach statistical significance (*P* = 0.13). No relationship between RGCL thickness and contact-lens-corrected RA data was observed (Kendall’s tau = −0.03, *P* = 0.81). The results for spectacles and contact lens corrected data are displayed in Figs. 6A and B respectively.

### Retinal ganglion cell number underlying Ricco’s area

**Figure 7:**
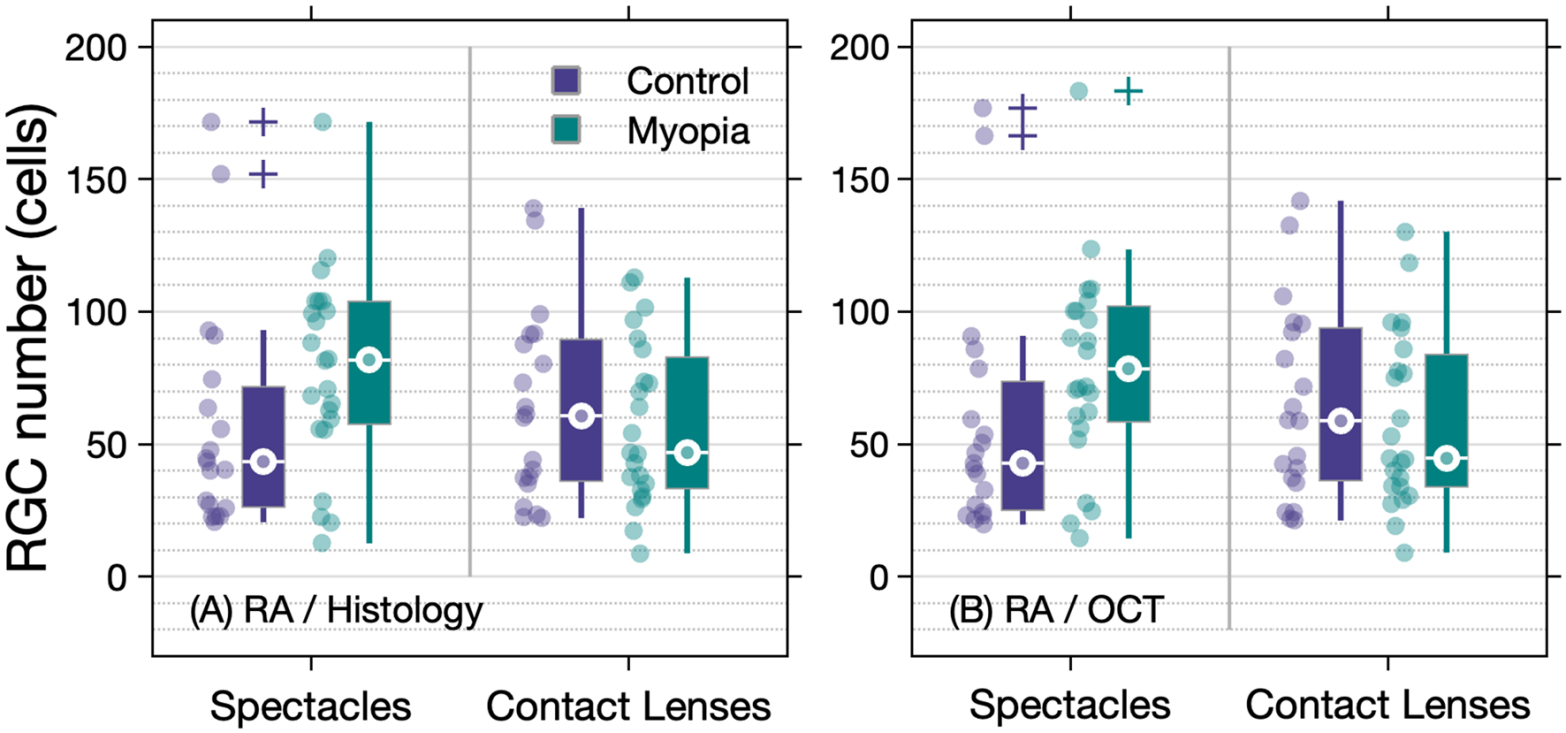
Boxplots reporting the number of RGCs underlying Ricco’s area in the control and myopia cohorts with spectacle and contact lens correction as estimated using **(A)** scaled histological data, and **(B)** OCT derived RGCL thickness values.

Using both methods of calculation, no statistically significant difference in RGC number underlying RA was observed between the myopia and control groups with spectacle or contact lens correction (histology method: Kruskal–Wallis ${\chi }^{2}$(3) = 6.3, P = 0.10; OCT method: Kruskal–Wallis ${\chi }^{2}$(3) = 6.6, P = 0.09). Despite this, estimates of RGC number underlying RA (median, IQR) were found to be higher in the myopia cohort (histology: 81.7 cells, IQR 57.6 to 103.9; OCT: 78.4 cells, IQR 58.4 to 102.1) compared to control observers (histology: 43.4 cells, IQR 26.3 to 71.7; OCT: 42.9 cells, IQR 25.1 to 73.7) when examined with spectacle correction (Fig. 7). Conversely, RGC number was lower in the myopia cohort (histology: 46.9 cells, IQR 33.3 to 82.8; OCT: 44.8 cells, IQR 33.9 to 83.8) compared to controls (histology: 60.8 cells, IQR 36.1 to 89.5; OCT: 58.9 cells, IQR 36.3 to 93.9) with contact lens correction.

## Discussion

When inter-observer differences in the projected retinal image size are controlled for (Knapp’s law invoked), peripheral RA was found to be larger in the myopia group compared to non-myopic controls. Such differences were not present when identical psychophysical measures were performed with contact lens correction where RIS varied proportionally with axial length (i.e., Knapp’s Law was not satisfied). To our knowledge, this is also the first study to observe a statistically significant, positive correlation between peripheral RA (spectacle corrected) and co-localized measurements of peripheral ocular length.

The finding of altered spatial summation in myopia is in agreement with the two previous studies that have investigated this topic. Jaworski et al.^35^ reported the foveal ‘critical area’ to be 0.16 log units larger in a high-myopia cohort (refractive error above −8.50DS) compared to non-myopic controls for an achromatic stimulus in the fovea. Atchison et al.^19^ also considered spatial summation in a large cohort both centrally and out to 30º along the horizontal meridian, the authors reporting a 0.03 log unit increase in RA per diopter increase in myopia. While such trends point towards altered spatial summation in axial myopia, differences in functional testing methodology and statistical analyses severely limit inter-study comparisons. For example, only the fovea was examined by Jaworski et al.^35^ compared with a region at 10º eccentricity in the current study, it being known that spatial summation varies with visual field eccentricity.^22,26^ Another key difference is the use of contrasting summary values to reflect the extent of spatial summation being exhibited. Jaworski et al.^35^ compared ‘critical area at maximum summation’ in myopes and non-myopic controls, defining this metric as the transition from partial summation to no summation. In the present study and that of Atchison et al.^19^, the upper limit of complete spatial summation (RA) was used to describe the extent of spatial summation. Furthermore, a constrained fitting technique was used by both Jaworski et al.^35^ and Atchison et al.^19^ to generate summation functions; a methodology which can lead to inaccuracies when extracting summary values from summation data^36^. Other inter-study differences include stimulus chromaticity, background luminance and psychophysical test setup (e.g., staircase step-size, auditory stimuli, etc.)

Other studies have previously presented evidence in support of changes in spatial vision, and by inference spatial summation, in myopia. For example, it has been reported that visual acuity^16,17^ and peripheral resolution acuity^4,18,19^ are reduced in myopia. Other work quantifying aniseikonia in participants with anisomyopia also provides evidence for altered spatial summation. Bradley and colleagues^37^ used a dichoptic size matching test with identical inter-eye RIS (i.e., Knapp’s law invoked) to reveal large degrees of residual aniseikonia (22%) with spectacle lens correction, such differences being proportional to the degree of axial elongation. Interestingly, in two observers with measures repeated with contact lens correction (where Knapp’s law did not hold) aniseikonia was markedly reduced (3.9%) in their study. Such results closely reflect the observations made in the present study whereby RA was related to axial length when RIS was optically controlled, this relationship not being apparent with CL correction. Bradley et al.^37^ propose that such findings may be related to perceptual minification of the retinal image in the myopic eye, potentially arising secondary to inter-eye differences in retinal stretching. Similar work undertaken by Rabin et al.^38^ proposed that axial anisometropia-induced aniseikonia reflects differences in the spatial density of ‘retinal elements’.

### Physiological basis of altered spatial summation in myopia

Much debate surrounds the physiological basis of spatial summation in the human visual system. It has been proposed that the density of retinal neurons (e.g., photoreceptors, RGCs)^31,55^, RGC receptive field organization^34,56,57^ and higher visual centers^55,58^ each contribute to the measured RA or ‘perceptive field’, with changes to the functional or structural integrity of these features potentially inducing alterations in spatial summation. Previous work examining photopic spatial summation in observers with no eye disease, found RA to enlarge as a function of visual field eccentricity^22^, this change being attributed to variations in the density of retinal neurons moderating stimulus detection. Work examining spatial summation in glaucoma reported similar changes to occur secondary to reductions in functional RGC density^31^, it being hypothesized that such alterations in spatial summation occur to maintain input to cortical receptive fields from a constant number of functionally intact RGCs, thus maintaining a constant signal-to-noise ratio. It has also been proposed^31,34,55^ that a fixed number of RGCs underlie RA across the visual field, accounting for changes in spatial summation area as a function of visual field eccentricity. In the case of the current study, it is possible that a similar hypothesis is applicable in myopia, where ocular growth and subsequent retinal stretch leads to reductions in localized RGC density^4,18,19^ and an enlarged RA serves to maintain a constant number of RGCs underlying RA and a constant signal–noise ratio for contrast detection. This hypothesis may be further supported by the fact we observed no statistically significant difference in RGC number estimated to underlie RA when modelled using both normative histological and OCT data (Fig. 7).

While considering changes in the density of RGCs in myopia as the sole source of alterations in RA is convenient, it is likely that multiple loci in the visual pathway play a role. For example, a strong relationship between co-localized RGCL thickness and RA would be expected if the density of RGCs was the sole factor determining the size of RA. However, in the present study only a weak negative relationship was observed between these variables, similar to previous findings relating RA to co-localized RGC number derived from psychophysical measures in glaucoma^31^. Furthermore, despite there being no statistically significant differences in estimated RGC number underlying RA in myopia and control participants, marked variability in these values was observed (Fig. 7). In the context of the myopic visual system, alterations in the density of RGCs^4,18,19^ and function of higher-visual centers^59,60^ have been reported previously, with changes in the organisation of RGC receptive fields also being hypothesized to occur in response to altered chemical balance in the body. For example, dopamine and dopamine antagonists are known to alter the balance between the center and surround components of center-surround antagonistic receptive fields of retinal neurons by altering the degree of electrical coupling between cells^61–65^. This role has been demonstrated in rabbit on-bipolar cells whereby dopamine concentration was increased in photopic conditions, leading to an increase in the weighting of the off-surround, whereas maintained darkness and/or blocking dopamine receptors led to diminished receptive field surrounds^66^. Looking specifically at RGCs, Jensen and Daw^67^ found dopamine antagonists to cause a reduction in the antagonistic surround input to the off-center RGC receptive field, leading to a shift in the center-surround arrangement in favour of the center (i.e., larger central receptive field size). Previous authors have proposed RA to be a psychophysical correlate of the relationship between RGC receptive field centre and surrounds in the retina^22,27,57^, with the potential that dopamine alters this balance and thus RA. Much evidence points towards reduced retinal dopamine levels in myopia^61,68,69^, with light exposure (which stimulates dopamine release in the retina^70^) associated with a reduction in myopia onset and progression^71–73^. It is therefore conceivable that the larger RA found in myopia may be a consequence of lower dopamine levels in this group.

Other work points to the role of the visual cortex in moderating spatial summation. Redmond et al.^58^ found changes in RA with background luminance for the S-cone pathway, where retinal center-surround receptive field organisation is known not to exist, the authors proposing this to point to the influence of higher visual centers. Indeed, a cortical contribution^22^ or basis^31,74,75^ to RA has been suggested by several authors. Such changes may take the form of alterations in the spatial tuning of cortical filters or an active remodelling of the visual cortex in response to changes in the density of retinal neurons as demonstrated in in vivo animal studies^76,77^. More recent functional MRI work has also identified altered structure^59^ and functional-connectivity deficits^78^ within the visual pathway of patients with high myopia. It is therefore possible that the changes in RA observed in this study may reflect changes to multiple loci of the visual pathway, including higher visual centres, in myopia.

Whilst the neurological underpinnings of RA are still debated, it is clear from this study and others^79,80^ that optical factors can also profoundly influence measurements of spatial summation. Specifically, it is evident that when optically induced changes in RIS, occurring secondary to axial elongation in myopia, are accounted for a perceptual ‘minification’ remains, manifesting as an enlarged RA in axial-myopes relative to controls. By contrast, such differences in RA were not observed when contact lens correction was used and Knapp’s Law not satisfied. In this instance, a lack of minifcation of the retinal image by the refractive correction leads to RIS proportionally increasing with axial elongation and RA being ‘filled’ more rapidly; this relationship breaking down when Knapp’s law is satisfied and RIS remains constant with axial length. Similar results were reported by Atchison et al.^19^ who observed a stronger relationship between RA and refractive error after post-hoc correction for inter-observer differences in RIS. This interplay between neural and optical factors is thought to account for residual perceptual aniseikonia in anisometropia when measured with a constant inter-eye RIS^37,38^. Indeed such findings may be a consequence of increased spatial summation in the axially myopic eye, these neural changes serving to compensate for an enlarged RIS and thus optimize visual function.

### Implications for the clinical assessment of spatial vision

The outcomes of the present work may have implications for both the assessment of spatial vision in observers with myopia, but also for the development and interpretation of tests of spatial vision designed to detect ophthalmic diseases (e.g., perimetry for glaucoma). Considering the association between ocular length and measures of RA observed in this study, it is possible that changes in RA may act as a non-invasive, functional marker of global or localized (i.e., equatorial or posterior pole elongation) globe expansion in progressive myopia^81^. For example, in the absence of biometric measures and concurrent disease, RA values may be measured at multiple locations and reflect the extent of local retinal stretch/axial elongation present when RIS is carefully controlled. Measurements of RA could also potentially be combined with structural measures in myopia (e.g., axial length, retinal thickness) to enable progressive myopia to be detected and monitored more robustly. Combining different sources of information, from both structural and functional measures, has been demonstrated to be more effective than considering just one clinical measure in isolation for other ocular conditions where monitoring and predicting progression is important (e.g., glaucoma, ocular hypertension)^82–84^.

The results of the present study also have potential implications for the design of perimetric test strategies used to detect functional deficits in glaucoma. Specifically, those tests (e.g., area-modulation perimetry^85^) intended to probe alterations in spatial summation in glaucoma may need to incorporate a normative database stratified according to AL if the balance between AL induced changes in RIS and neural minification is not maintained (i.e., spectacle lens used to correct refractive error) in axial myopes. Incorporating such information will serve to increase the specificity of such a test to detect *true* glaucoma related changes in RA and not those secondary to axial expansion of the globe.

## Conclusions

In summary, our novel observation of an increased RA in axial-myopia when RIS is invariant of AL suggests spatial summation to be altered in the myopic, but otherwise healthy, visual system. We propose that this finding represents a functional adaptation of the myopic visual system to an enlarged RIS in the axially-elongated globe. The implications of this research are three-fold in that it, (i) builds our knowledge of the structure/function relationship in myopia, (ii) provides ‘normal myopic control’ information for similar research in glaucoma, and (iii) creates the potential for the development of a non-invasive functional test for myopic progression. Further work is however necessary to determine if the ratio of measurement variability to changes in RA in myopia (i.e., myopia signal-to-noise ratio) is favourable across all stages of myopia.

## Supplementary information

Supplementary file1 (PDF 1521 kb)

## Data Availability

Supporting data will be made available upon request from the corresponding author.
