# Supplementary material for: Altered spatial summation optimizes visual function in axial myopia
Source: Sci Rep. 2020 Jul 22;10:12179. doi: 10.1038/s41598-020-67893-8 (PMC7376210; doi:10.1038/s41598-020-67893-8)
Supplement: Supplementary file 1 — Supplementary file1 (PDF 1521 kb) [file 41598_2020_67893_MOESM1_ESM.pdf]

Supplementary material for:

ALTERED SPATIAL SUMMATION OPTIMIZES VISUAL FUNCTION IN AXIAL MYOPIA

Victoria Stapley,[1] Roger S. Anderson,[1,2] Kathryn J. Saunders,[1] Pádraig J. Mulholland*[1, 2]

[1] Centre for Optometry and Vision Science, Biomedical Sciences Research Institute, Ulster University, Coleraine, UK

[2] National Institute for Health Research (NIHR) Biomedical Research Centre at Moorfields Eye Hospital NHS Foundation Trust and UCL Institute of Ophthalmology, London, UK

Methods to estimate retinal ganglion cell number underlying Ricco's Area

The number of retinal ganglion cells (RGC) underlying Ricco's Area (RA) was estimated using two methods in this study. In method one alterations in RGC density were modelled with changes in participant axial length through a global expansion ('balloon') model of myopic eye growth assuming a constant number of RGCs in the eye (histology model), with method two using the method of Raza and Hood[1] whereby OCT derived RGCL thickness is integrated with histological data to provide patient specific local estimates of RGC volumetric density and number (OCT model). Common to both methods was the use of RGC counts (RGC/mm^2) reported by Curcio and Allen[2] for five, young observers (six healthy eyes, mean age 33.8 years). As this data was however only presented for the horizontal and vertical meridians (see their fig. 6), it was first necessary to extrapolate RGC density values across the central retina. This was performed using a method similar to that reported by Garway-Heath et al.[3] and Raza and Hood,[1] whereby the raw histological data within the central 4 mm of the retina along the superior, inferior, nasal and temporal meridians was linearly interpolated along polar coordinates to generate estimates of RGC/mm^2 at 10,000 locations across the central retina (fig. S1). Considering the histological data of Curcio and Allen[2] is presented with the fovea and optic nerve on the horizontal meridian (fig. S2-A), the normative RGC/mm^2 data sets produced were rotated according to the OCT measured angular subtense between the macula and optic nerve head centre in each observer for use in both models (fig. S2-B).

For all calculations, the abbreviated axial length method of Bennett et al.[4] was used to produce an observer specific conversion factor (q_p) to translate degrees in visual space to mm on the retina. Briefly, this required the conversion factor (q) to be calculated for the fovea ($q_0 = 0.01306*[\textit{axial length}-1.82]$), with an alteration being made for the eccentricity (U, in degrees) at which the functional measures were performed ($q_p = q_0-0.000014U^2$). Using q_p the eccentricity in degrees

(ecc_{deg}, eq.1) at which functional measures were captured, in addition to stimulus area (eq. 2, where Φ is stimulus diameter in degrees) were converted to mm and mm^2 on the retina respectively.

$$\text{ecc}_{mm} = ecc_{deg} . q_p \quad \text{[eq. 1]}$$

$$\text{S}_{area} = \pi . \left(\frac{\Phi}{2} . q_p \right)^2 \quad \text{[eq. 2]}$$

In the case of spectacle correction, whereby Knapp's Law was invoked and inter-observer retinal image size (RIS) was constant in mm on the retina, an axial length value equal to that expected in an emmetropic eye (23.3 mm) was used for the calculation of q_p. Conversely, the *true* axial length value was input for each observer when considering contact lens correction, such that q_p varied with globe expansion. The effect of scaling stimulus area and test eccentricity under the conditions of spectacle and contact lens correction are presented for axial length values between 21-31 mm in fig. S2-B.

Both the histology and OCT methods for the calculation of RGC number are described in detail below.

1. Histology method

The extrapolated histological RGC/mm^2 values were proportionally scaled according to the degree to which each observers axial length varied from the mean value expected in an emmetropic observer[5] (scaling factor = 23.3/*axial length*). This step served to simulate alterations in RGC density/mm^2 expected in a simple global expansion model of myopia where the total RGC number remains constant, but local RGC density is uniformly reduced secondary to retinal stretch (fig. S3). RGC density (RGC/mm^2) over the corresponding region of stimulus presentation was then

extrapolated through linear interpolation in two-dimensions from the RGC density map generated from histological data. The number of RGCs underlying a given Ricco's area stimulus (RGC_{Hist}) was subsequently calculated as the product of the mean sampled histologically derived normative RGC/mm^2 values and stimulus area in mm^2.

2. OCT method

This first required a measure of retinal ganglion cell layer (RGCL) thickness (μm) in the retinal location corresponding to functional measures to be extracted from the posterior pole OCT scans in each participant. In this study, RGCL thickness was averaged over individual 3°x3° Spectralis measurement grid squares within which the corresponding visual field locations fell after correction for lateral RGC displacement from underlying photoreceptors.[6] RGC density (RGC/mm^2) was then linearly interpolated in two-dimensions over the area of the RA stimulus from the extrapolated histological RGC density map. Unlike the histology method, values were however not proportionally scaled with participant axial length. The number of RGCs underlying the RA stimulus (RGC_{OCT}) was then calculated in each observer using equation 3.

$$\mathrm{RGC}_{OCT} = RGCL \cdot GCD \cdot \mathrm{S}_{area} \qquad \text{[eq. 3]}$$

Where $RGCL$ is OCT derived RGCL thickness in mm, GCD is co-localized RGC volumetric density (RGC/mm^3) calculated by dividing the mean RGC/mm^2 across the area of the stimulus (derived from histological data) with mean co-localized RGCL thickness values from a group of healthy observers (mm, expressed as mean of RGCL thicknesses within defined grid square), and S_{area} the area of the RA scaled stimulus in mm^2. Outputs of both the histology and OCT methods for the estimation of RGC number underlying a 0.43° diameter stimulus and RA stimuli for the control and myopia observers in this study are illustrated in fig. S4.

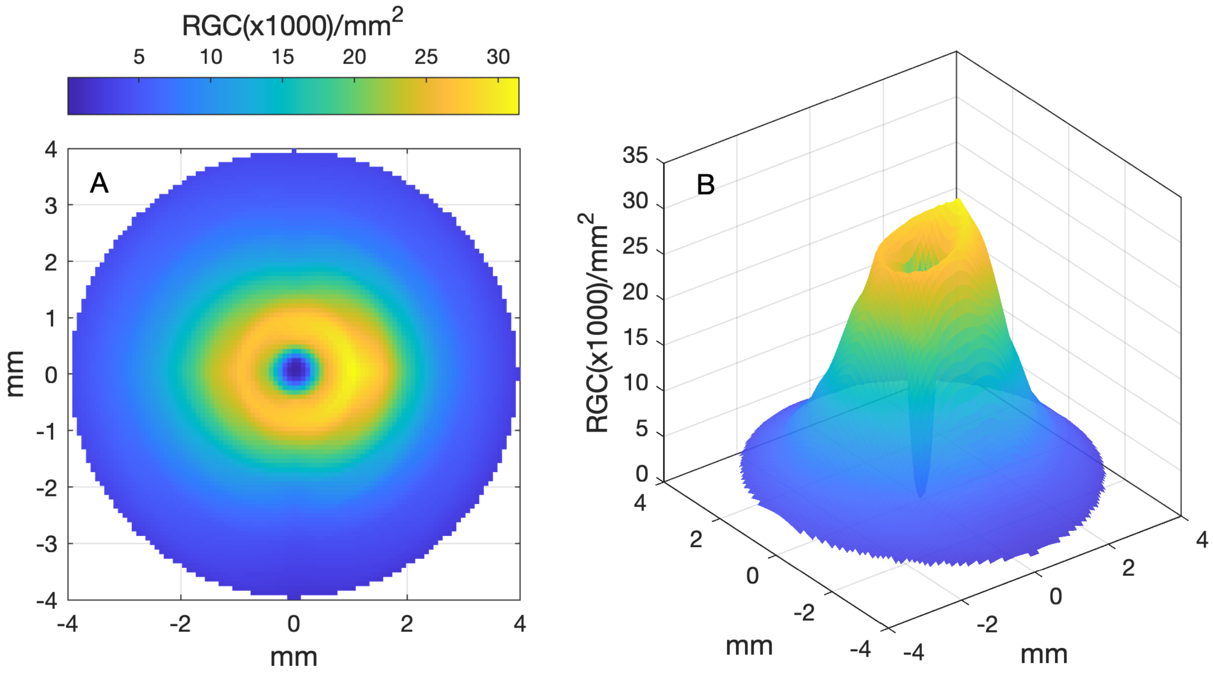

Fig. S1: Extrapolated histological RGC density per mm^2 values based on the data of Curcio and Allen[2] presented as two- (A) and three-dimensional forms (B). (Figure plotted with MATLAB R2016a, The MathWorks Inc., USA, www.mathworks.com)

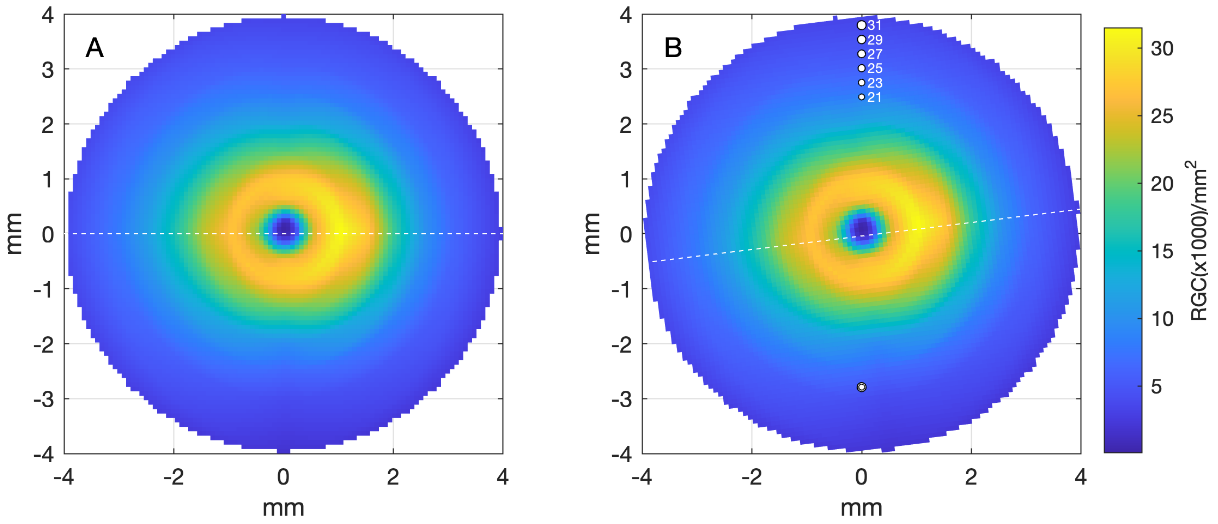

<u>Fig. S2</u>: (A) En-face RGC density plot illustrating original orientation of histological data with fovea and optic nerve head on horizontal meridian (white dashed line). (B) Example adjustment in orientation of extrapolated histological data for angular subtense between fovea and optic nerve head (7°, dashed white line). Also included for reference are projected retinal image sizes for a 0.43° diameter stimulus in observers with a range of axial lengths (21-31 mm) under the conditions of contact lens wear (superior retina) and spectacle correction where Knapp's Law is satisfied (inferior retina). (Figure plotted with MATLAB R2016a, The MathWorks Inc., USA, www.mathworks.com)

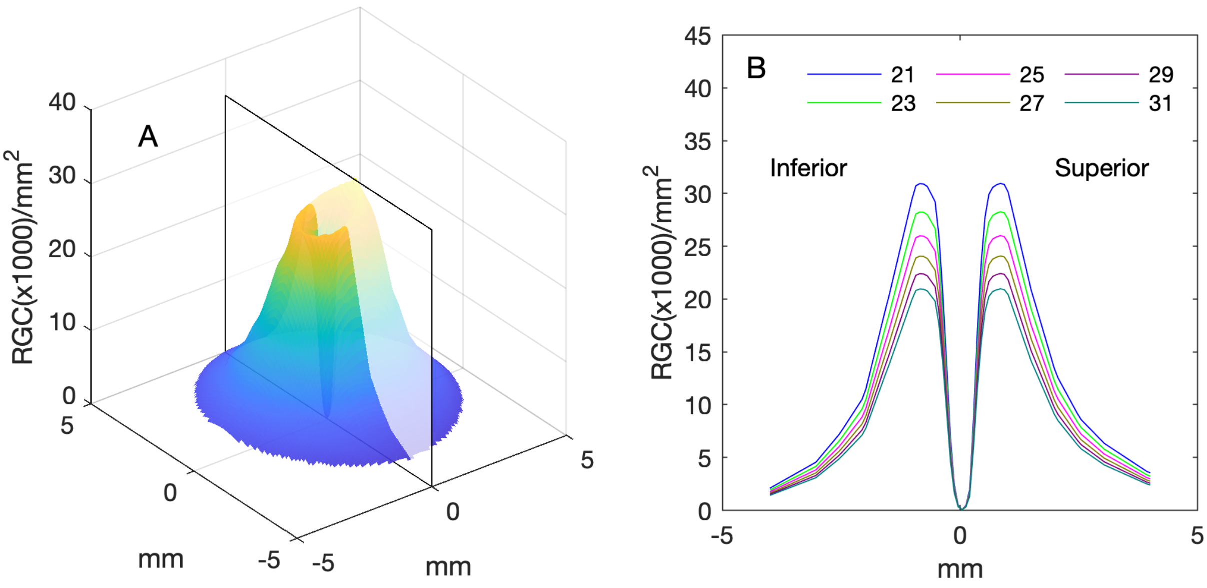

Fig. S3: Plots of (A) RGC density/mm^2 at locations within the central retina extrapolated from histological RGC counts in healthy eyes[2], and (B) RGC density profile along vertical meridian (translucent plane in A) demonstrating a range of simulated variations in RGC density/mm^2 with changes in axial length (21-31 mm) assuming a uniform expansion of the globe and a constant number of RGCs. (Figure plotted with MATLAB R2016a, The MathWorks Inc., USA, www.mathworks.com)

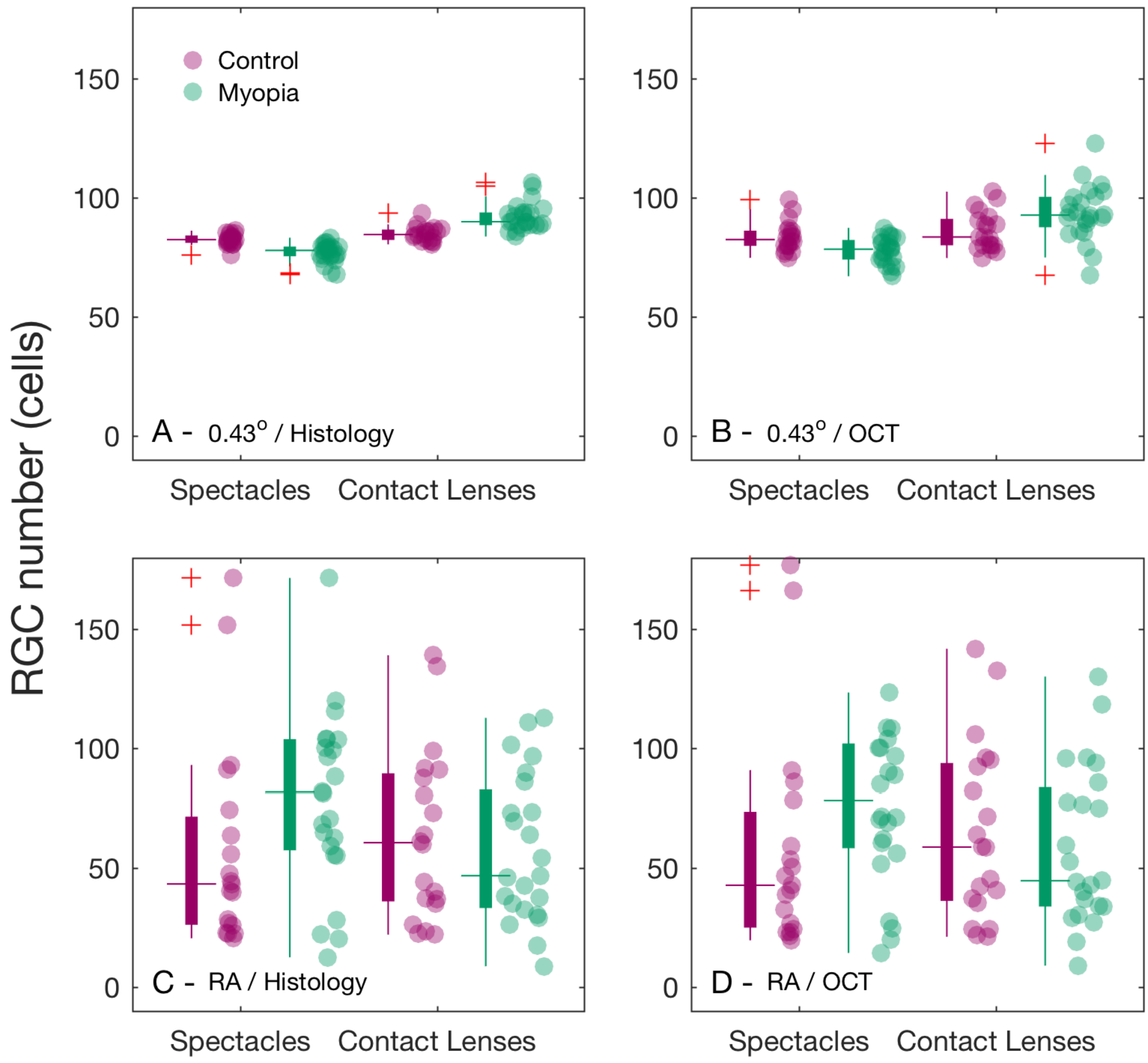

Figure S4: Number of RGCs estimated to underlie (A) a 0.43° diameter stimulus and (B) Ricco's Area in the control and myopia cohorts with spectacle and contact lens correction.

References

1 Raza, A. S. & Hood, D. C. Evaluation of the Structure-Function Relationship in Glaucoma Using a Novel Method for Estimating the Number of Retinal Ganglion Cells in the Human Retina. *Invest Ophthalmol Vis Sci* **56**, 5548-5556, doi:10.1167/iovs.14-16366 (2015).
2 Curcio, C. A. & Allen, K. A. Topography of ganglion cells in human retina. *J Comp Neurol* **300**, 5-25, doi:10.1002/cne.903000103 (1990).
3 Garway-Heath, D. F., Caprioli, J., Fitzke, F. W. & Hitchings, R. A. Scaling the hill of vision: the physiological relationship between light sensitivity and ganglion cell numbers. *Invest Ophthalmol Vis Sci* **41**, 1774-1782 (2000).
4 Bennett, A. G., Rudnicka, A. R. & Edgar, D. F. Improvements on Littmann's method of determining the size of retinal features by fundus photography. *Graefes Arch Clin Exp Ophthalmol* **232**, 361-367 (1994).
5 Atchison, D. A. *et al.* Eye shape in emmetropia and myopia. *Invest Ophthalmol Vis Sci* **45**, 3380-3386, doi:10.1167/iovs.04-0292 (2004).
6 Drasdo, N., Millican, C. L., Katholi, C. R. & Curcio, C. A. The length of Henle fibers in the human retina and a model of ganglion receptive field density in the visual field. *Vision Res* **47**, 2901-2911, doi:S0042-6989(07)00006-5 [pii]
10.1016/j.visres.2007.01.007 (2007).
